# Refinement of bamboo genome annotations through integrative analyses of transcriptomic and epigenomic data

**DOI:** 10.1016/j.csbj.2021.04.068

**Published:** 2021-04-30

**Authors:** Xuelian Ma, Hansheng Zhao, Hengyu Yan, Minghao Sheng, Yaxin Cao, Kebin Yang, Hao Xu, Wenying Xu, Zhimin Gao, Zhen Su

**Affiliations:** a State Key Laboratory of Plant Physiology and Biochemistry, College of Biological Sciences, China Agricultural University, Beijing 100193, China; b Key Laboratory of National Forestry and Grassland Administration/Beijing for Bamboo & Rattan Science and Technology, Institute of Gene Science and Industrialization for Bamboo and Rattan Resources, International Center for Bamboo and Rattan, Beijing 100102, China; c College of Agronomy, Qingdao Agricultural University, Qingdao, Shandong, China

**Keywords:** Moso bamboo, Genome annotation, Transcriptomic data, Epigenomic data, H3K4me3

## Abstract

Bamboo, one of the most crucial nontimber forest resources worldwide, has the capacity for rapid growth. In recent years, the genome of moso bamboo (*Phyllostachys edulis*) has been decoded, and a large amount of transcriptome data has been published. In this study, we generated the genome-wide profiles of the histone modification H3K4me3 in leaf, stem, and root tissues of bamboo. The trends in the distribution patterns were similar to those in rice. We developed a processing pipeline for predicting novel transcripts to refine the structural annotation of the genome using H3K4me3 ChIP-seq data and 29 RNA-seq datasets. As a result, 12,460 novel transcripts were predicted in the bamboo genome. Compared with the transcripts in the newly released version 2.0 of the bamboo genome, these novel transcripts are tissue-specific and shorter, and most have a single exon. Some representative novel transcripts were validated by semiquantitative RT-PCR and qRT-PCR analyses. Furthermore, we put these novel transcripts back into the ChIP-seq analysis pipeline and discovered that the percentages of H3K4me3 in genic elements were increased. Overall, this work integrated transcriptomic data and epigenomic data to refine the annotation of the genome in order to discover more functional genes and study bamboo growth and development, and the application of this predicted pipeline may help refine the structural annotation of the genome in other species.

## Graphical abstract

**Figure:**
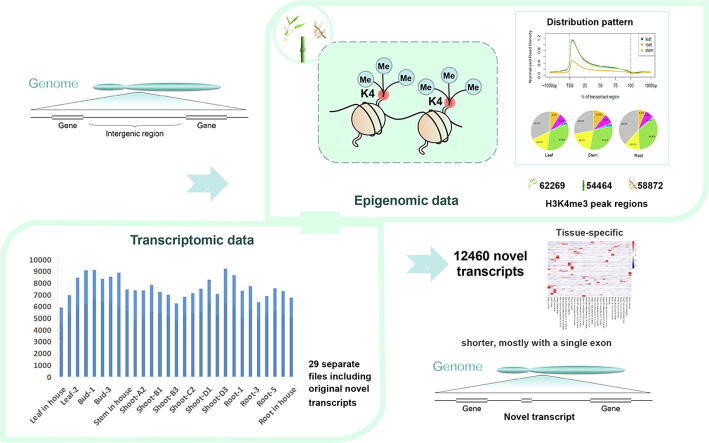

## 1 Introduction

Bamboo, a perennial grass, is one of the most crucial nontimber forest resources worldwide. Its rapid growth ability has increasingly attracted people’s attention to its economical use, ecology and culture [1], [2], [3], [4], [5], [6], [7], [8], [9], [10], [11]. Moso bamboo (*Phyllostachys edulis*), which is grown on over two-thirds of the total bamboo growing area (4.68 million hectares) [12] in China, is a promising bioresource for renewable nonwood forestry products; a complete draft genome was annotated in 2013 and an updated chromosome-level reference genome (version 2.0) in 2018 [2], [13]. However, the structural annotation of the latest bamboo genome still needs a great deal of improvement based on more supporting evidence.

The dynamic structure of chromatin and various functions of genes rely on multiple epigenetic mechanisms, most notably posttranslational modification of histones [14], [15]. In eukaryotes, the lysine residues at the N-terminus of histone H3 or H4 can be mono-, di-, or trimethylated, and these modifications play an important role in the maintenance of normal transcription patterns and the regulation of gene expression [16], [17]. H3K4 trimethylation (H3K4me3), an evolutionarily conserved epigenetic mark, is enriched exclusively within the promoters and 5′ end of transcribed regions with a 5′-to-3′ gradient along genes in animals and plants [16], [18], [19]. In the plant kingdom, genome-wide analyses of the epigenetic mark H3K4me3 have been performed in several species, including *Arabidopsis* [16], [20], rice [19], [21], [22], [23], maize [24], tomato [25], potato [26], cotton [27], [28] and *Brachypodium distachyon* [29], and the results suggest that H3K4me3 is usually located ~150 bp upstream to ~500 bp downstream of transcription start sites (TSSs). Therefore, H3K4me3 could be a marker for the identification of novel transcripts and genes and the improved annotation of genome structure [27].

There are a number of methods and workflows for the structural and functional annotation of whole draft genome sequences, operating through three main analytical strategies: *de novo* prediction [30], homology-based prediction and transcriptome-based prediction [31], [32], [33], [34]. Some famous institutes and projects, such as NCBI, Ensembl, Broad and BGI, have developed their own annotation systems to provide genome annotation services [35]. In addition, some well-known software programs have been developed for whole-genome annotation, for example, MARKER2 [36] and BRAKER [37]. Specifically, in humans or other model species, with the help of BLAST software, we can find ORFs that encode protein sequences similar to those in databases or find them *de novo* without reference to cDNA sequences. Available transcriptomic and epigenomic data make genome annotation more efficient and comprehensive through novel transcript prediction methods in plants. For instance, histone modification data within the genomic and transcriptomic data for multicellular organisms have been employed in predictions of alternative splicing patterns in *Gossypium hirsutum* and *Gossypium arboretum* [27].

For bamboo, large amounts of transcriptome data from various tissues across different growth stages [2] have become available to support the prediction of potentially unknown transcripts, especially these tissue-specific transcripts. Bamboo epigenomic data including H3K4me3 data will also help in the prediction and discovery of novel transcripts across diverse tissues. Here, we successfully determined the genome-wide landscape of the histone modification marker H3K4me3 in three tissues of bamboo by the ChIP-seq method to refine the structural and functional annotation of the bamboo genome.

## 2 Material and methods

### 2.1 Plant material and growth conditions

Moso bamboo (*Phyllostachys edulis*) plants were grown in pots filled with soil for three months, which maintained under the following conditions: 28 °C, 16/8 h of light/darkness, and relative humidity of 50%. The leaf, stem, and root samples were harvested after thorough rinsing with clean water, respectively, and then preserved in liquid nitrogen for ChIP-seq and RNA-seq.

### 2.2 ChIP-seq and data analysis

Chromatin immunoprecipitation (ChIP) experiments were performed using anti-trimethyl-histone H3 (Lys 4) (H3K4me3, Millipore, 07-473) as described previously [38]. Approximately 15 g of leaf, stem, and root tissues were collected to undertake ChIP experiments and sequencing, respectively. Bowtie2 software [39] was used to align the sequencing reads of ChIP-seq to the bamboo reference genome version 2.0 [2] using default parameters. The H3K4me3 deposition peaks were called by MACS v1.4.1 program [40]. The no-model parameter and the d-value parameter at 200 were set. The resulting wiggle files, which represent counts of ChIP-seq reads across the reference genome, were normalized for sequencing depth by dividing the read counts in each bin by the millions of mapped reads in each sample and were visualized in the UCSC genome browser [41]. The CEAS software [42] was used to analyze the distance between TSSs of genes and the nearest called peaks. H3K4me3 peaks located in the region 2 kb upstream of TSSs and gene bodies were considered H3K4me3-associated genes. The differential regions showing H3K4me3 modifications between two of three tissues were also called by MACS v1.4.1 program with the d-value parameter of 200. We then identified genes with differentially changed H3K4me3 peaks located in the region 2 kb upstream of TSSs and gene bodies.

### 2.3 RNA-seq and data analysis

Total RNA was extracted using TRIzol reagent (Invitrogen) and purified using RNeasy Mini Kits (Qiagen). RNA of three tissues (leaf, stem, and root) with the same source as the ChIP experiments were isolated and sequenced. Sequencing reads of RNA-seq were aligned to the bamboo reference genome version 2.0 [2] (including the novel predicted transcripts or not) using TopHat software [43]. Genes showing statistically significant differential expression on the basis of (log_2_ fold change >0.6) were identified as DEGs by using Cuffdiff in Cufflinks packages [34]. R packages were used to do hierarchical analysis using novel predicted transcripts.

### 2.4 qRT-PCR

Extracted total RNA was reverse transcribed using an M-MLV kit (Invitrogen). The samples, 10 µl each containing 2 µg of total RNA and 20 pmol of random hexamers (Invitrogen), were maintained at 70 °C for 10 min to denature the RNA and then chilled on ice for 2 min. The reaction buffer and M-MLV enzyme (20 µl of the mixture contained 500 µM dNTPs, 50 mM Tris-HCl (pH 8.3), 75 mM KCl, 3 mM MgCl_2_, 5 mM dithiothreitol, and 200 units of M-MLV) was added to the chilled samples, and the samples were maintained at 37 °C for 1 h. The cDNA samples were diluted to 8 ng/μL for qRT-PCR analysis.

qRT-PCR assays were performed in triplicate on 1 µl of each cDNA dilution using the SYBR Green Master Mix and an ECO Real-Time PCR system (Illumina) according to the manufacturer’s protocol. The amplification of 18S rRNA was used as an internal control to normalize all data (forward primer, 5′-CGGCTACCACATCCAAGGAA-3′; reverse primer, 5′-TGTCACTACCTCCCCGTGTCA-3′). The transcript-specific primers of semiquantitative RT-PCR and qRT-PCR are listed in Supplemental Table 4. The relative quantification method (ΔΔCT) [44] was used for quantitative evaluation of the variation between replicates.

### 2.5 An integrative approach to identify novel transcripts

Twenty-nine transcriptomic datasets were used to identify novel transcripts for the first step. Twenty-six of them were obtained from National Center for Biotechnology Information (NCBI) Short Read Archive (SRA) database, and the accession numbers are SRX2408703-28 [2]. The other three transcriptomic datasets were got in this study. All of them were derived from sequencing variety *Phyllostachys edulis*. TopHat [43], Cufflinks [34], Bedtools [45], custom scripts, and Cuffmerge were utilized in order to predict putative novel transcripts. With the adoption of TopHat, the files of bam (the binary version of a SAM file) format were got to predict novel transcripts by means of Cufflinks packages, and the files of GTF format were further integrated through Bedtools and custom scripts. Then, H3K4me3 peaks were predicted by MACS and CEAS using epigenomic datasets. Next, the final novel transcripts have been identified through the co-occurrence analysis of merged transcripts and distributed H3K4me3 peaks. In addition, the histone modification peaks with peak centers near the novel transcripts (<2000 bp) were proposed to identify the transcript strand. When the center was close to the left end of a novel transcript, then the strand was forward; when the center was near the right end, then the strand was reverse. H3K4me3 peaks from leaf, stem and root tissues were used independently. Lastly, the coding ability and functional annotation of novel transcripts were assessed by Blastn program (E-value <1.0E^−5^) against moso bamboo cDNA sequences and by Blastx program (E-value <1.0E^−3^) against the protein sequences from the UniprotKB database. The online and standalone version of Coding Potential Calculator (CPC2) [46] prediction were also useful and helpful for the coding ability.

## 3 Results

### 3.1 Global analysis of trimethylation of histone H3 lysine 4 (H3K4me3) marker in bamboo

The genome-wide landscape of the histone modification H3K4me3 in bamboo was measured by ChIP-seq in this study. In addition, tissue samples were used for mRNA-seq experiments in parallel to perform an auxiliary investigation of the characteristics of H3K4me3 in three tissues: leaf, stem and root. By quality control and then aligning ChIP-seq reads to the version 2.0 reference genome of moso bamboo (*Phyllostachys edulis*) using Bowtie2 software, most reads (from 95.29% to 96.43%) were mapped to the genome (Table 1), which suggested that the sequencing data were of high quality. The RNA-seq data of bamboo were also mapped to version 2.0 of the genome using TopHat, and the overall read mapping rates of all three tissues were higher than 85% (Supplemental Table 1). *Oryza sativa*, from the grass family (Poaceae), has a close relationship with bamboo, and the characteristics of H3K4me3 have been reported. The rice H3K4me3 ChIP-seq datasets were obtained from previous studies [23], and the read mapping rates were 97.41% and 91.17% in leaves and roots, respectively (Supplemental Table 2). Therefore, we compared these rice datasets along with bamboo ChIP-seq datasets in this study.

**Table 1:** Mapping information of H3K4me3 ChIP-seq datasets in leaf, stem and root tissues of moso bamboo.

Histone modification	Species	Tissue	Total reads	Overall alignment rate	Aligned exactly 1 time	Aligned > 1 times	Peaks	Genes with peaks
H3K4me3	Bamboo	Leaf	30,896,249	96.42%	36.59%	59.82%	62,269	39,523
		Stem	31,326,042	96.43%	37.80%	58.63%	58,872	39,178
		Root	24,857,577	95.29%	27.13%	68.16%	54,464	34,384

Histone modification peaks were called through MACS (model-based analysis using ChIP-seq) and CEAS software. Then, we performed a comparison between the distribution pattern of H3K4me3 in the three tissues and the gene density. H3K4me3 peaks were found more frequently in high-gene density regions at the whole-genome level (Fig. S1). There were more H3K4me3 peaks on the chromosome ends, and the gene densities in these regions were high.

**Fig. 1:**
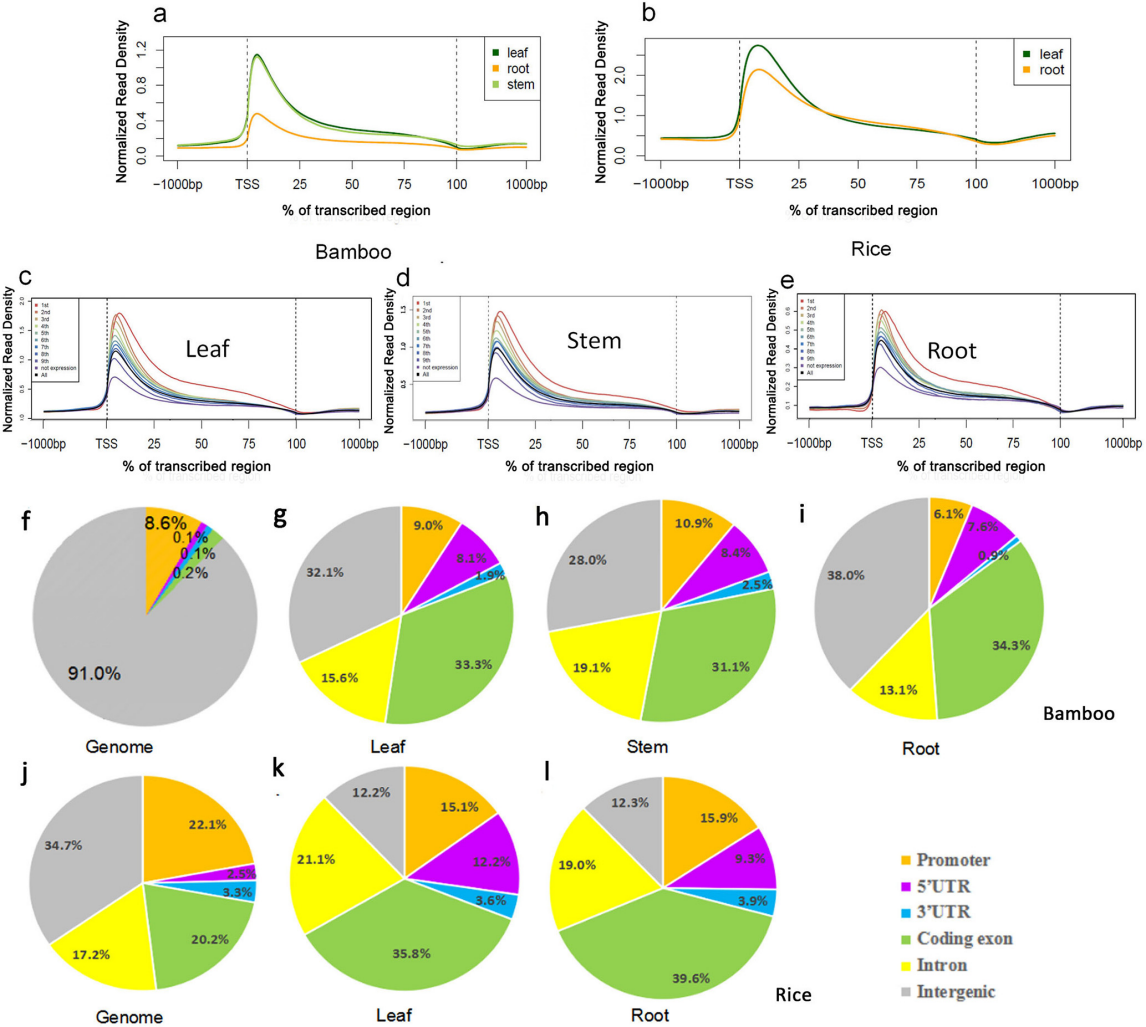
Characterization of the H3K4me3 distribution pattern in bamboo and rice. (a, b) The distribution of H3K4me3 along all genes in the leaves, stems and roots of bamboo (from 1 kb upstream to 1 kb downstream) compared to its relative *Oryza sativa* (rice). (c–e) The meta-gene profile of H3K4me3 among genes with different expression levels in bamboo leaves, stems and roots. Genes (FPKM > 0) were divided into nine quartiles based on their expression levels, and nonexpressed genes (FPKM = 0) were marked as ‘not expressed’. (f and j) Percentages of six regional classes (*i.e.,* promoter, 5′ untranslated region (UTR), 3′ UTR, coding exon, intron, and intergenic region) in the bamboo and rice genomes. (g–i) Genomic distribution of H3K4me3 peaks within different regions in bamboo leaves, stems and roots. (k and l) Genomic distribution of H3K4me3 peaks within different regions in rice leaves and roots. The bamboo and rice genomes were classified into six categories: five genic regions (promoter, 5′ UTR, 3′ UTR, coding exon and intron) and intergenic regions.

Then, we normalized the sequencing reads along the genic regions to obtain meta-gene profiles of H3K4me3, which showed that H3K4me3 was significantly enriched at the transcription initiation region in the three tissues of bamboo. As expected, this distribution pattern around gene bodies in bamboo was mostly similar to that described in a previous report in rice [19], [21], [22] (Fig. 1a, b), indicating that our results were convincing. The deposition pattern of H3K4me3 at the TSS regions was conserved in bamboo, as it is in plants [16], [18], [19], [20], [24], [25], [26], [27], [29], whereas the distribution of H3K4me3 modification had different degrees of tissue specificity. Although the signal intensities of H3K4me3 showed little difference in the leaves and stems of bamboo, they were higher than those in the roots. In addition, we further analyzed the relationship between histone modification and gene expression levels to explore the role of H3K4me3 in the regulation of gene transcription in bamboo. We observed that H3K4me3 was more likely to be highly deposited at the TSSs of the genes with high expression levels. There was a positive correlation between the H3K4me3 modification and gene expression levels in all three tissues of bamboo (Fig. 1c–e). Genes with high expression levels had a tendency to show a high histone modification level, suggesting that H3K4me3 is correlated with active transcripts and is a conserved histone marker in several model plants [16], [18], [19], [20], [24], [25], [26], [27], [29].

We identified the total peaks of histone modification H3K4me3 deposition. Approximately 60,000 H3K4me3 peaks were identified in bamboo, and there were more peaks (62,269) in leaves than in roots (54,464) or stems (58,872) (Table 1). Most H3K4me3 peaks were conserved in different tissues of bamboo, whereas a few may be tissue-specific. Subsequently, 39,523, 39,178, and 34,384 bamboo genes with H3K4me3 peaks were distinguished in leaves, stems and roots, respectively. We identified 25,334 genes with H3K4me3 from 26,199 peaks in the leaves of rice and 30,873 genes from 32,820 peaks in the roots of rice (Supplemental Table 2). In contrast, there were many more overlapping regions of H3K4me3 and transcripts in rice, suggesting that these epigenomic datasets could be an available resource for improving structural genome annotation and predicting missing genes in bamboo.

Next, we analyzed the genome-wide distribution of H3K4me3 based on the regions related to genes, including the promoter, 5′ untranslated region (5′ UTR), 3′ UTR, exon, intron and intergenic region (Fig. 1f–l). There were some similarities in the H3K4me3 peak distribution in bamboo leaves, stems, and roots, one of which was that H3K4me3 peaks were mainly enriched in genic regions (promoter, 5′ UTR, 3′ UTR, exon and intron) in the three tissues. The coding exonic regions with H3K4me3 deposition accounted for approximately one-third of the whole-genome regions in each of the three tissues. Next, the intronic areas with H3K4me3 peaks in leaves, stems and roots of bamboo accounted for 15.6%, 19.1% and 13.1% of the global regions, respectively. Finally, the percentages of promoter, 5′ UTR and 3′ UTR regions with H3K4me3 peaks were fairly low. For example, the percentage of 3′ UTR regions with H3K4me3 peaks in root tissue was only 0.9%. In contrast, intergenic regions accounted for the largest share (91.0%) of the bamboo genome. The percentages of intergenic regions with H3K4me3 deposition among leaves, stems and roots of bamboo were relatively high, up to 32.1%, 28.0% and 38%, respectively (Fig. 1f–i).

Regarding the distribution of H3K4me3 in rice, intergenic regions accounted for merely 34.7% of the genome, which was much lower than the 91.0% in bamboo. The percentage of intergenic regions containing H3K4me3 modification peaks in leaf and root tissues of rice was only approximately 12% (Fig. 1j–l), and the percentages of the promoter, 5′ and 3′ UTR, exonic, and intronic regions with H3K4me3 deposition in leaf and root tissues of rice were consistently higher than those in bamboo regardless of the tissue. Thus, the higher proportion of intergenic regions with H3K4me3 deposition may indicate that the annotation of the moso bamboo genome could be further improved by the identification of novel genes in unknown genomic segments and intergenic regions.

### 3.2 Identification of novel transcripts in the annotated bamboo genome with transcriptomic profiles and epigenomic maps

**Fig. 2:**
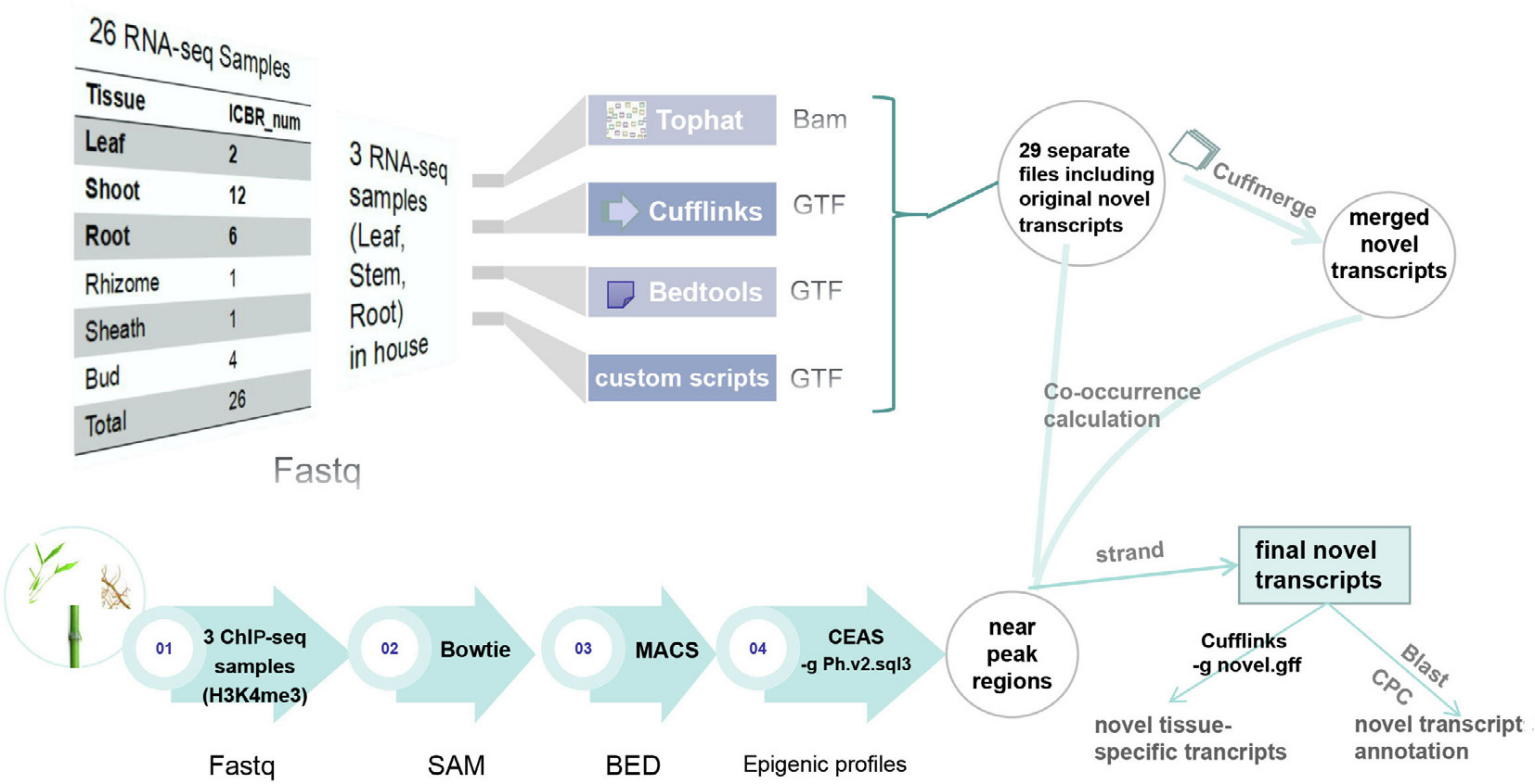
A flowchart depicting the processing pipeline for predicting novel transcripts in bamboo.

A previous study [27] used groups of RNA-seq datasets to build gene models and identify potential exonic regions in plant genomes. Combined with the pre-existing analysis methods and tools for novel transcript discovery, we constructed an analysis pipeline for the identification of novel transcripts in the latest annotated bamboo genome (Fig. 2). To comprehensively predict putative novel transcripts, we used three in-house and 26 public transcriptomic datasets covering different portions of leaf, shoot, root, rhizome, sheath, and bud tissues during different developmental stages [2], prompting us to scan and search for as many novel transcripts as possible. As a result, we obtained 29 independent files containing the original novel transcripts, which were merged into 19,767 novel transcripts (Supplemental Table 3). In addition, we added the H3K4me3 peak-containing regions in leaves, stems and roots from the above analyses of three epigenomic datasets to increase the credibility of the novel transcript predictions. Finally, the novel transcripts were identified and annotated based on co-occurrence analysis of merged transcripts and distributed H3K4me3 peaks. The detailed prediction process is described in the methods section.

**Fig. 3:**
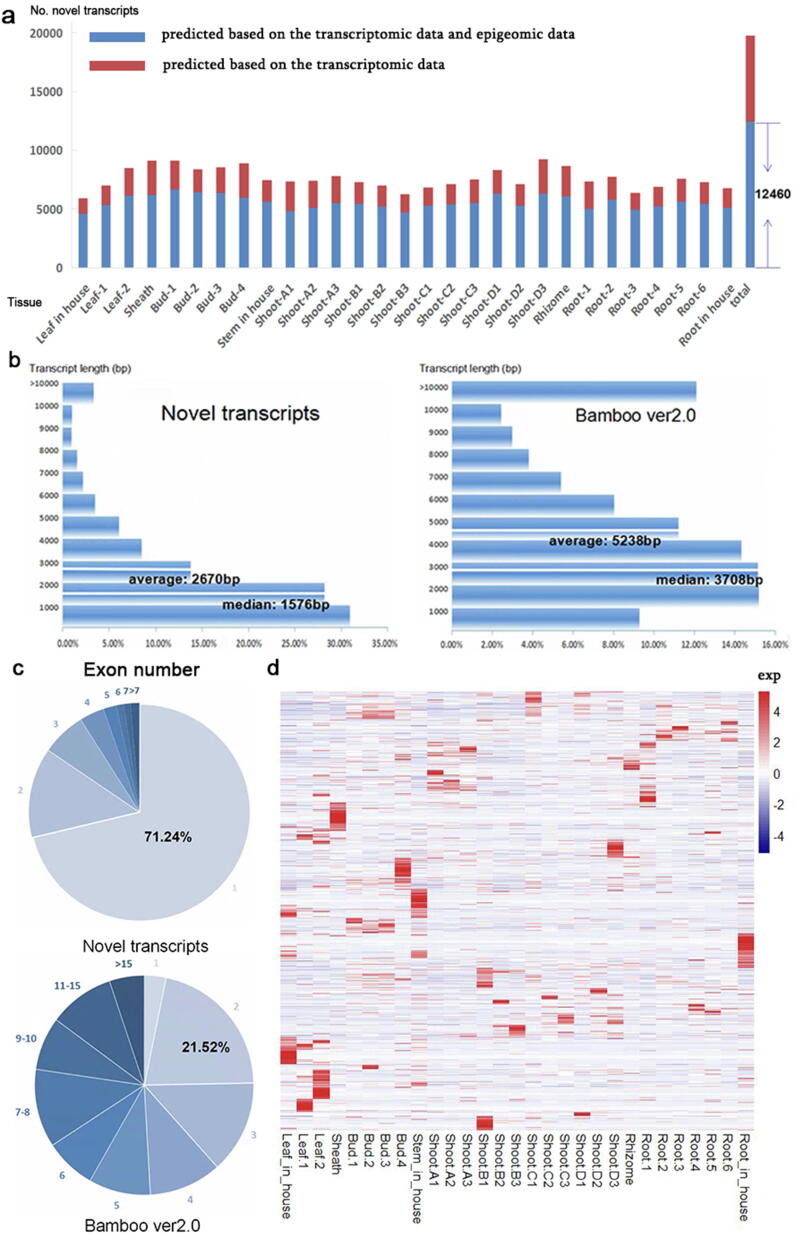
Statistics for the novel transcripts and comparison with the latest structural annotation of the bamboo genome. (a) Summary of novel transcripts in the annotated bamboo genome with transcriptomic data and epigenetic maps. The shade of blue represents the number of overlaps between the total number of novel transcripts in bamboo derived from RNA-seq data and the H3K4me3 peak-containing regions in leaves, stems, or roots. (b) Comparison of the transcript length between novel transcripts and transcripts in version 2.0 of the bamboo genome. (c) Comparison of the exon number between novel transcripts and transcripts in version 2.0 of the bamboo genome. (d) Heatmap clustering of newly identified transcripts among different RNA-seq samples. (For interpretation of the references to color in this figure legend, the reader is referred to the web version of this article.)

We performed a combination of ChIP-seq and related RNA-seq datasets. Taking tissue leaves as an example, we predicted 5935 novel transcripts, and the numbers of transcripts overlapping with H3K4me3 peaks in leaf, stem, and root tissues and any one of them were 4443, 4469, 3826 and 4588, respectively (Supplemental Table 3). Thus, 12,460 novel predicted transcripts were merged in the predicted results, and these transcripts also overlapped with H3K4me3 peak regions in leaf, stem or root tissue at the same time (Fig. 3a). A total of 9040 novel transcripts in the bamboo RNA-seq datasets also showed evidence of H3K4me3 in the three tissues (leaf, stem and root) (Fig. S2), whereas 380, 570 and 229 merged novel transcripts in the RNA-seq datasets were supported in these respective three tissues, which suggested that these transcripts may be tissue-specific.

We further compared the transcript length and exon number between novel transcripts and the latest annotation. We found that the newly predicted transcripts were shorter (Fig. 3b). For example, the newly predicted transcripts were mostly 1000–2000 bp in length with median and average lengths of 1576 bp and 2670 bp, respectively, while the transcripts in the latest annotation were mostly 2000–5000 bp in length with median and average lengths of 3708 bp and 5238 bp, respectively. High-quality annotations showed that the transcript length in the plant model *Arabidopsis* was at most 1000–2000 bp, while the transcript length in rice was mostly within 1000 bp (Fig. S3). Furthermore, 71.24% of the newly predicted transcripts were single-exon transcripts, and those in the latest annotation were mostly multiexon transcripts, most (21.52%) of which contained two exons (Fig. 3c).

We presented the distribution of the 12,460 newly predicted transcripts in the 29 transcriptomic datasets. Many transcripts were not expressed or were expressed in only one or a few tissues, while 37.5% of the transcripts in the latest annotation were expressed in 29 tissues (Fig. S4). Additionally, the average expression levels of the newly predicted transcripts in 29 tissues were lower than those in the latest released annotation (Fig. S5). We also showed clustered expression patterns of the newly predicted transcripts (Fig. 3d), which revealed that multiple novel transcripts were usually expressed in specific tissues. However, most genes in the latest annotation were expressed in various tissues. More precise genome-wide structural annotation will contribute to research on unknown functional gene regulation among different tissues in bamboo.

**Fig. 4:**
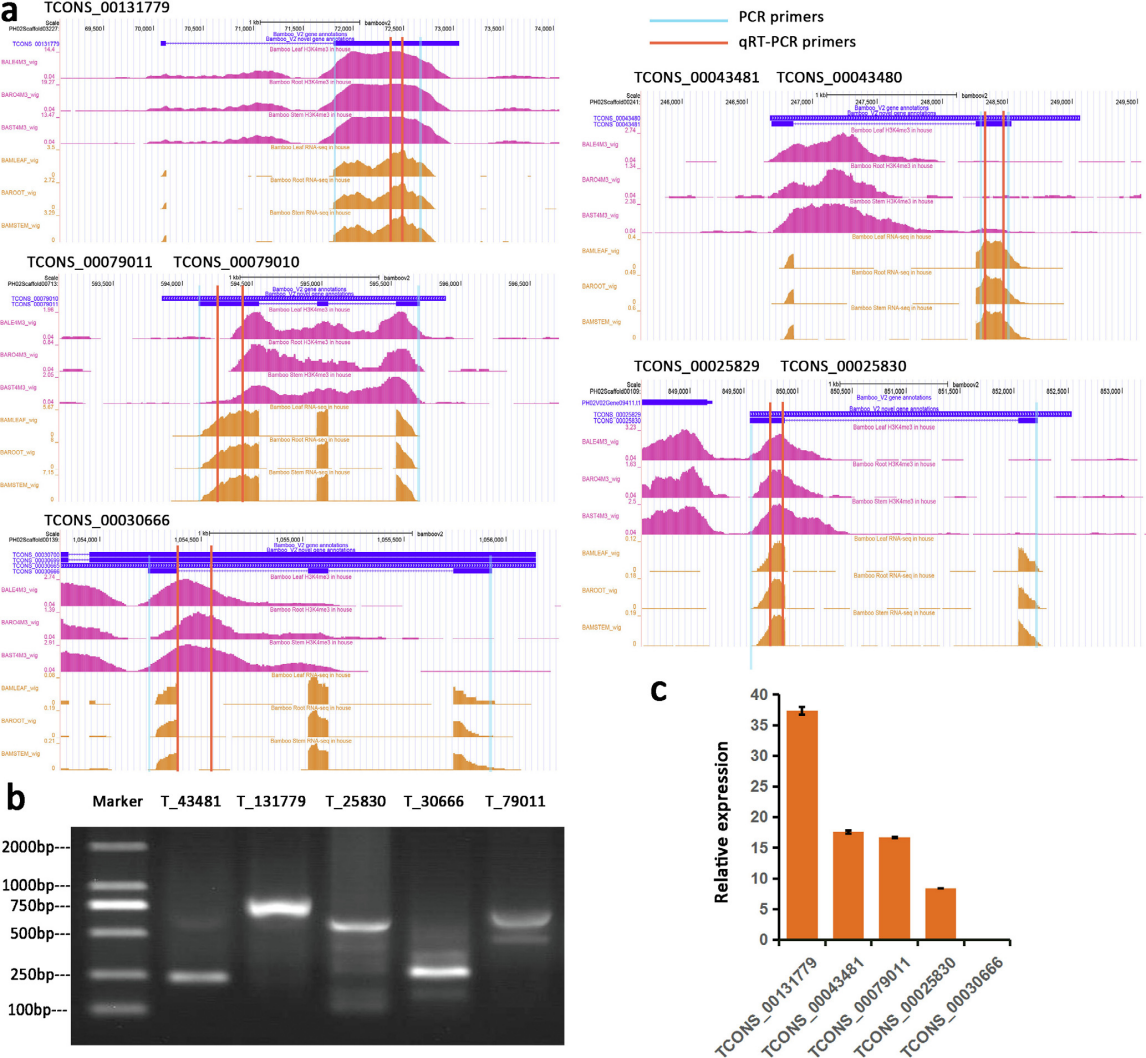
UCSC Genome Browser and semiquantitative RT-PCR analyses of representative novel predicted transcripts. (a) UCSC Genome Browser screenshots of TCONS_00131779, TCONS_00043481, TCONS_00043480, TCONS_00079011, TCONS_00079010, TCONS_00025829 and TCONS_00025830. The purple and orange peaks represent the H3K4me3 deposition and expression levels of novel transcripts, respectively. (b) Agarose gel electrophoresis of semiquantitative RT-PCR products for five novel predicted transcripts of bamboo from the mixture of leaf, stem and root tissues. Lanes: Marker, molecular size marker; T_43481, TCONS_00043481; T_131779, TCONS_00131779; T_25830, TCONS_00025830; T_30666, TCONS_00030666; T_79011, TCONS_00079011. (c) Relative expression levels of TCONS_00131779, TCONS_00043481, TCONS_00079011, TCONS_00025830 and TCONS_00030666 in stems of bamboo were obtained by qRT-PCR. The amplification primer for TCONS_00030666 was located in the intronic region as the negative control. (For interpretation of the references to color in this figure legend, the reader is referred to the web version of this article.)

We visualized the novel transcripts with the UCSC Genome Browser, which has a convenient and intuitive interface to visualize the gene structure of putative transcripts, as well as the corresponding expression pattern and H3K4me3 peak distributions in all RNA-seq and ChIP-seq datasets. Five selected novel transcripts, including TCONS_00131779 and TCONS_00043481, are shown as examples in the screenshot from the UCSC Genome Browser (Fig. 4a). Each of them had peaks calculated from the corresponding three in-house RNA-seq datasets, representing the unidentified exons, as well as peaks calculated from the three ChIP-seq datasets, which revealed that they were expressed in all three tissues (leaf, stem and root). The semiquantitative RT-PCR and qRT-PCR results demonstrated the accuracy of the novel transcript predictions (Fig. 4b, c). The best hit for the novel transcript TCONS_00025830 in *Arabidopsis thaliana* was *AT2G02510* (E-value = 5e^−06^), whose encoded protein might act as a NADH dehydrogenase (ubiquinone). The best hit for another novel transcript, TCONS_00079011, in *Arabidopsis thaliana* was *MT2A* (*AT3G09390*, E-value = 9e^−13^), whose encoded protein binds to and detoxifies excess copper and other metals, limiting oxidative damage. Thus, the predicted pipeline has proven, to some extent, the possibility and feasibility of refining the current gene annotation.

### 3.3 Comparative analysis of H3K4me3 deposition among bamboo leaf, stem and root tissues based on the inclusion of novel predicted transcripts

**Fig. 5:**
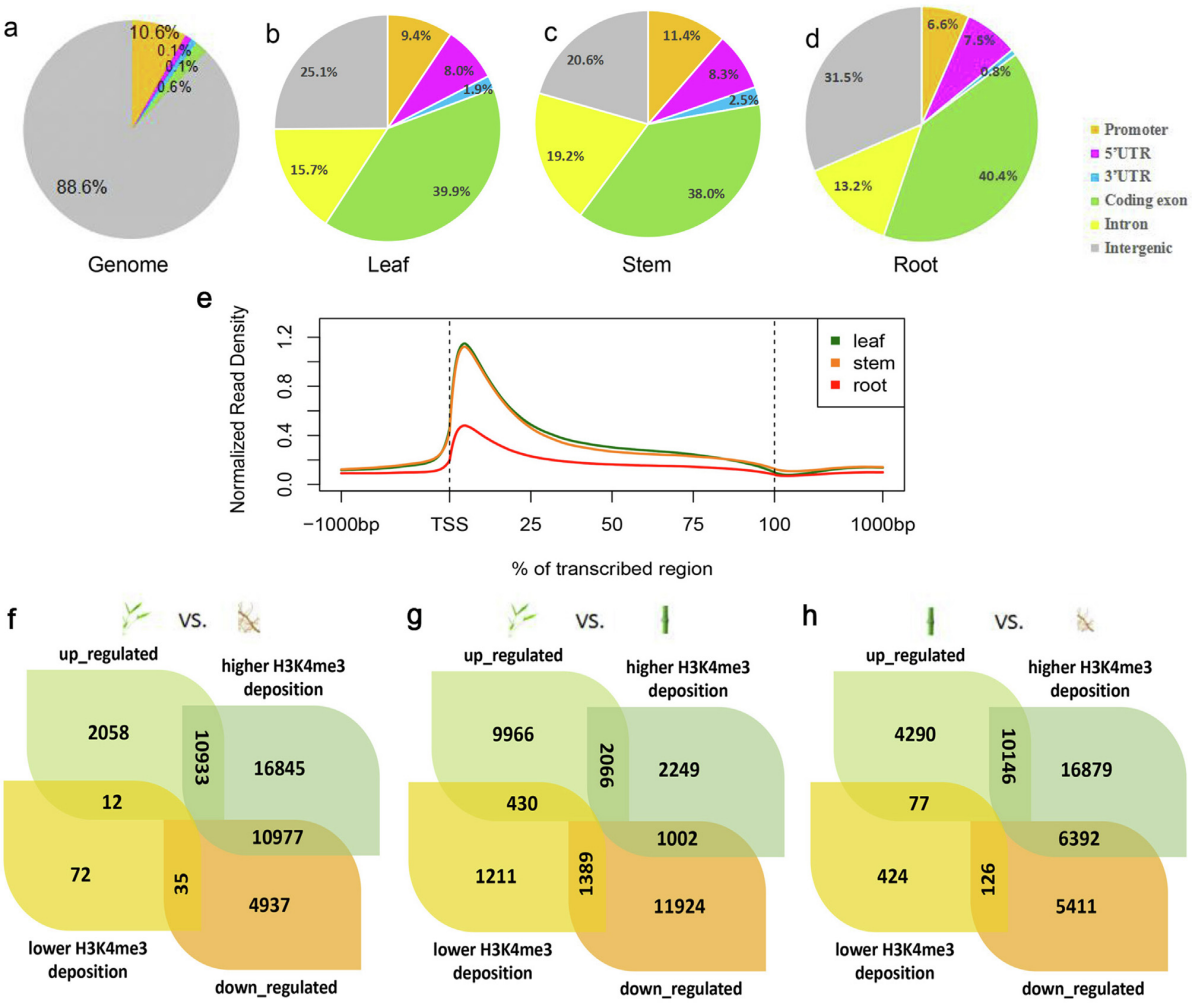
Comparative analysis of genes with H3K4me3 deposition among leaf, stem and root tissues based on the inclusion of novel predicted transcripts combined with their expression levels. (a) Percentages of the known and novel predicted bamboo transcripts in the six regional classes [promoter, 5′ untranslated region (UTR), 3′ UTR, coding exon, intron and intergenic region]. (b–d) Genomic distribution of H3K4me3 peaks within different regions in bamboo leaves, stems and roots based on the inclusion of novel predicted transcripts. (e) The distribution of H3K4me3 along known and novel predicted bamboo transcripts in the leaves, stems and roots. (f–h) Venn diagram showing the overlap of genes with different H3K4me3 deposition patterns and genes differentially expressed between two of the three tissues (leaf, stem, and root).

We reannotated the genome-wide distribution of H3K4me3 within different regions in the latest released annotation (version 2.0) and an updated annotation with novel predicted transcripts for bamboo. The percentage of intergenic regions within the genome background of bamboo decreased from more than 90% in genome version 2.0 to 88.6%, and the percentages of intergenic regions with H3K4me3 deposition among leaves, stems and roots of bamboo decreased to 25.1%, 20.6% and 31.5%, respectively (Fig. 5a–d), which suggested that the protocol was feasible for predicting novel transcripts and improving genome annotation. Furthermore, the distribution trend of H3K4me3 among the three tissues based on the updated annotation was the same as that in version 2.0 (Fig. 5e). The numbers of genes with greater H3K4me3 deposition and higher expression levels in leaves *vs*. roots and stems *vs.* roots were higher than the numbers of genes with greater H3K4me3 deposition and higher expression levels in roots *vs.* leaves and roots *vs.* stems.

Based on the conclusion of novel predicted transcripts in bamboo, we proceeded to perform a paired comparison of the genes with H3K4me3 deposition in every pair of the three tissues (leaf, stem and root). Through integrated analysis, we identified 13,003 genes that were more highly expressed in leaves than in roots, while 15,949 genes were more highly expressed in roots. Moreover, 12,462 genes had higher expression levels in leaves than in stems, whereas 14,315 genes had lower expression levels in leaves. Ultimately, 14,513 genes were identified to have higher expression levels in stems than in roots, and 11,929 genes were expressed at lower levels in stems.

To investigate a potential relationship between gene expression and H3K4me3 enrichment, we performed overlap analysis of differentially expressed genes (DEGs) and genes with differentially distributed H3K4me3 peaks in different tissues. Comparison of H3K4me3 peaks between leaves and roots showed that 10,933 genes that had greater H3K4me3 depositions were upregulated in leaves, whereas 10,977 genes were upregulated in roots. Additionally, large amounts of photosynthesis-related genes were included among these 10,933 genes. Among the genes with higher H3K4me3 deposition in roots, 35 genes were upregulated in roots, but 12 genes were upregulated in leaves (Fig. 5f).

The same comparative analyses were performed for leaves *vs*. stems and stems *vs*. roots. Combined with the expression levels, there were 2066 genes with higher H3K4me3 peaks in leaves than in stems and were upregulated in leaves *vs.* stems, while there were 1389 genes with lower H3K4me3 peaks in leaves than in stems and were downregulated in leaves versus stems (Fig. 5g). Regarding stems versus roots, 10,146 genes with higher H3K4me3 deposition were upregulated, though 126 genes with lower H3K4me3 deposition were downregulated (Fig. 5h). Interestingly, some of these upregulated DEGs in leaves and roots (10,933 genes) that were related to photosynthesis were also among the upregulated DEGs in leaves and stems (2066 genes), suggesting that the genes with higher H3K4me3 deposition in leaves than in roots/stems, such as *LHCAs* and *LHCBs* (*PH02V02Gene46325*, *PH02V02Gene49542*, *PH02V02Gene27143* and *PH02V02Gene07024*), had higher expression levels in leaves than in roots/stems.

**Fig. 6:**
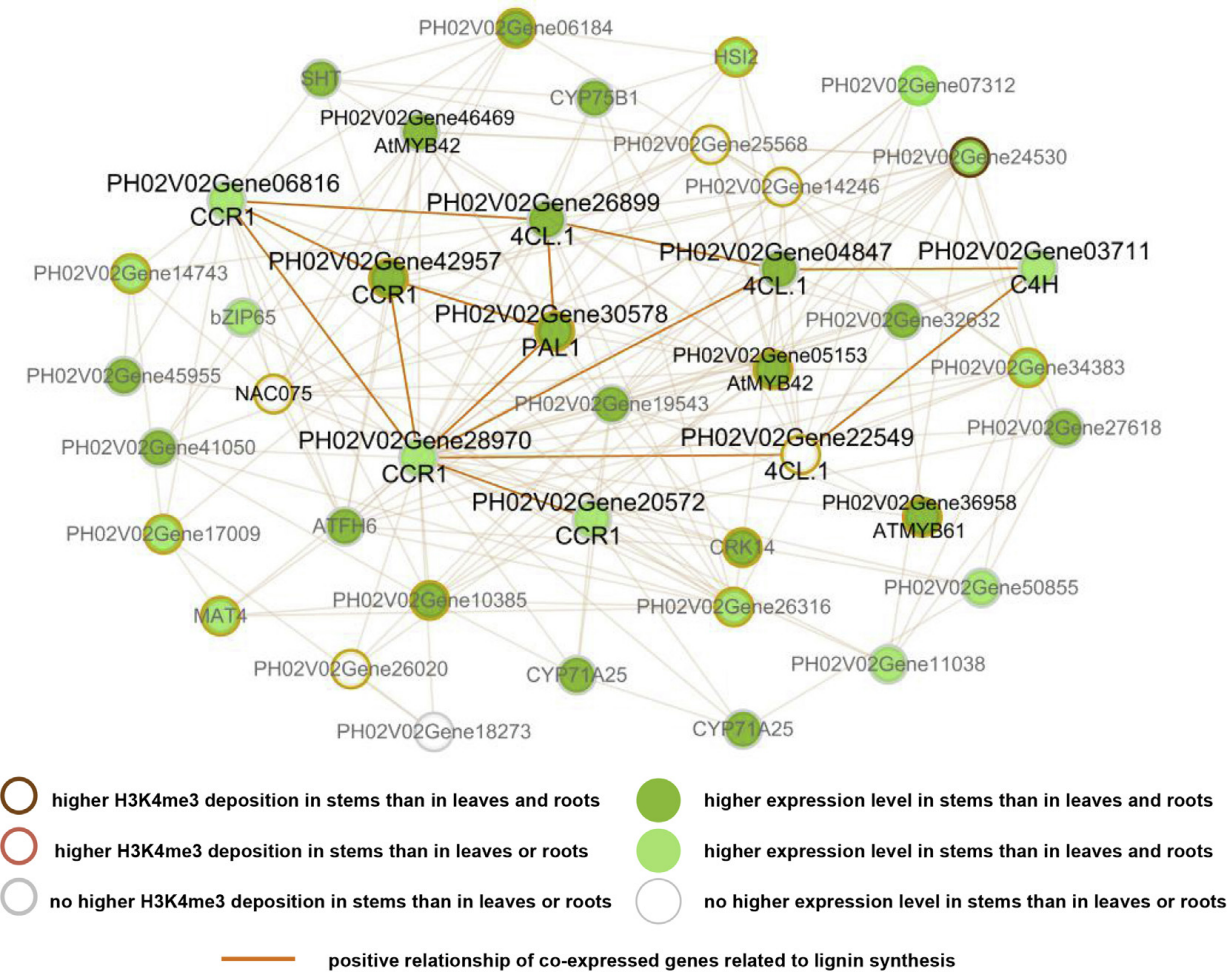
Coexpression network of genes related to secondary wall biosynthesis. The nodes in different colors represent genes with differential expression levels in stems versus leaves and/or roots. The genes with differential H3K4me3 deposition have different color boundaries. The edges connecting genes represent their positive relationship, and the thick brown lines are the edges of lignin biosynthesis genes. (For interpretation of the references to color in this figure legend, the reader is referred to the web version of this article.)

Based on these DEGs in stems, we further focused on their association with the phenylpropanoid pathway, which is essential for the fast growth and development of bamboo. The coexpression network constructed by the BambooNET database [47] showed that most of the genes were expressed at higher levels in stems than in leaves or roots and were involved in the phenylpropanoid pathway (Fig. 6). Additionally, some of these genes showed higher H3K4me3 deposition in stems than in leaves or roots, especially the genes related to phenylpropanoid biosynthesis *PH02V02Gene30578* (orthologous gene of *PAL1*), *PH02V02Gene42957* (orthologous gene of *CCR1*), an ortholog of AtMYB42 in bamboo (*PH02V02Gene05153)* and an ortholog of *AtMYB61* in bamboo (*PH02V02Gene36958*), which might be potentially involved in the rapid growth of bamboo, based on the correlation of H3K4me3 with active transcription in plants [16], [19], [29].

On the other hand, we further compared H3K4me3 deposition in different tissues. The regions around the TSSs in all bamboo transcripts and the novel predicted transcripts were clustered and divided into six clusters (Fig. S6). All six clusters showed similar trends in the three tissues, whereas the H3K4me3 signal was highest in leaves. Transcripts in cluster 1 and cluster 4 were enriched downstream and upstream of the TSSs, respectively. Additionally, the transcripts in the third cluster were distributed around TSSs, which indicated that the distribution trends of H3K4me3 among leaves, stems and roots were consistent with the trends of the H3K4me3 peaks shown in Fig. 5e.

### 3.4 Query and visualization of the novel predicted bamboo transcripts on a website

We uploaded detailed information on the novel predicted transcripts based on the latest bamboo annotation to BambooNET website (http://bioinformatics.cau.edu.cn/bamboo/) to browse and search for novel transcripts for researchers and users (Fig. S7a, b). During searching, if a newly predicted transcript ID was input into the search box, the predicted annotation, including the location of the start and end sites and the expression level, was provided (Fig. S7c). Additionally, the transcripts can also be shown by the UCSC Genome Browser for clear display within the genome (Fig. S7e). Visualization by the UCSC Genome Browser also showed H3K4me3 deposition in the three tissues (leaf, stem and root), as did all 29 RNA-seq data files used in the prediction of the novel transcripts. In addition, the information of all 12,460 novel transcripts can be browsed individually through the “Novel transcript browser” setting (Fig. S7b). If users are interested in some novel transcripts on the browser page, they can follow the links to the detailed information pages of these transcripts (Fig. S7d). Collectively, the query and visualization tools on the website will provide researchers with a comprehensive, convenient and intuitive way to utilize the novel predicted bamboo transcripts and explore their potential roles in bamboo growth and development.

## 4 Discussion

In this study, the epigenetic landscape of the histone modification marker H3K4me3 was determined in bamboo. We deciphered the conserved H3K4me3 distribution patterns in bamboo. The preliminary conclusions demonstrated that H3K4me3 was mainly distributed in genic regions and was enriched around TSSs, highly consistent with the patterns in other plants that already have epigenomic maps [16], [18], [19], [20], [24], [25], [26], [27], [29]. The histone marker was again shown to be associated with transcriptional activation in bamboo [15], [19], [27]. In addition, we identified several genes related to phenylpropanoid biosynthesis with modifications associated with higher H3K4me3 deposition and higher expression levels in stems versus leaves/roots. The presence of H3K4me3 ChIP-seq datasets in bamboo might provide an epigenomic perspective to study the genes potentially involved in the rapid growth and development of bamboo. Furthermore, the novel transcript TCONS_00034333 was only expressed in specific tissues at specific stages, *i.e.*, in the top portion of the 3 m shoot (Shoot-C1), and was likely related to shoot growth and development in bamboo; this finding provided insight into the research and applications of more putative transcripts. The discovery of novel transcripts facilitates the understanding of the regulation of tissue-specific genes to support the multiple stages of the rapid growth and development of bamboo.

A previous study reported that existing genome-wide annotation software or pipelines based mainly on RNA-seq data have low accuracy (20%–40%) for structural annotation of non-model genomes [48]. Compared with transcripts from version 2.0 of the bamboo genome, most of the novel transcripts are shorter and only have one exon, which may be one of the reasons for the low accuracy. However, the low accuracy may also be the result of a combination of complex factors. The novel transcripts were expressed at lower levels in most tissues and at higher levels in only one or a few tissues and may be tissue-specific transcripts. These characteristics of novel transcripts may have made them difficult to identify during previous genome-wide annotations or have caused them to be filtered out due to general thresholds. On the other hand, the distribution of single-exon transcripts may be species-specific. For example, single-exon transcripts accounted for a higher percentage (26.36%) of the genome in the model plant *Arabidopsis thaliana*, than in rice (only 1.94%) (Supplemental Tables 4 and 5). In summary, the discovery of the novel transcripts from ChIP-seq data will be beneficial to improve the prediction rate of single-exon transcripts and the accuracy of the bamboo annotation, and this pipeline is also applicable to other plant species for enhancing the accuracy of their genome annotation.

Accurate prediction of the novel transcript structure in bamboo requires the identification of more promoter-related markers, such as RNA polymerase II (Pol II), the identification of DNase I hypersensitive (DH) sites, and the results of cap analysis of gene expression (CAGE), as well as ChIP-seq data of markers positively correlated with transcription, such as H3K27ac. In contrast, the histone markers associated with transcriptional repression, such as H3K27me3 and H3K9me2 will make the prediction of transcript strand and tissue-specific expression patterns more convincing. In addition, a fraction of H3K4me3 peaks were too long, even covering the whole putative transcript, to define the novel transcript strand. The quantity and quality of RNA-seq data also influence the number and length of putative predicted transcripts. These defects and limitations will be compensated for and polished in the novel transcript prediction processing pipeline in the future as rising epigenome and transcriptome datasets are published. Notably, strand-specific RNA-seq datasets appear to be a dominant method to achieve accurate prediction of transcripts. Some transcripts erroneously merged in the pipeline will be divided more accurately, and the direction of the strand will be assessed more accurately. Alternatively spliced transcripts will be fully explored to evaluate the complexity of the genome structure, and precise expression levels of these novel transcripts will be completely acquired. On the other hand, the replaced tools HISAT2 [49] and StringTie [50] are expected to produce different predictions for the novel transcripts in bamboo. Hence, increasing attempts via diverse tools will render the detection of novel transcripts in bamboo more meaningful and comprehensive.

## Declaration of Competing Interest

The authors declare that they have no known competing financial interests or personal relationships that could have appeared to influence the work reported in this paper.
