# Supplementary material for: Refinement of bamboo genome annotations through integrative analyses of transcriptomic and epigenomic data
Source: Comput Struct Biotechnol J. 2021 Apr 30;19:2708–18. doi: 10.1016/j.csbj.2021.04.068 (PMC8131310; doi:10.1016/j.csbj.2021.04.068)
Supplement: Supplementary data 1 [file mmc1.docx]

**The refinement of bamboo genome annotations through the integrative analyses of transcriptomic and epigenomic data**

Xuelian Ma^†1^, Hansheng Zhao^†2^, Hengyu Yan^†3^, Minghao Sheng^1^, Yaxin Cao^1^, Kebin Yang^2^, Hao Xu^2^, Wenying Xu^*1^, Zhimin Gao^*2^, Zhen Su^*1^

^1^State Key Laboratory of Plant Physiology and Biochemistry, College of Biological Sciences, China Agricultural University, Beijing 100193, China.
^2^Key Laboratory of National Forestry and Grassland Administration/Beijing for Bamboo & Rattan Science and Technology, Institute of Gene Science and Industrialization for Bamboo and Rattan Resources, International Center for Bamboo and Rattan, Beijing 100102, China.
^3^College of Agronomy, Qingdao Agricultural University, Qingdao, Shandong, China.

^†^These authors contributed equally to this work.

*To whom correspondence should be addressed.


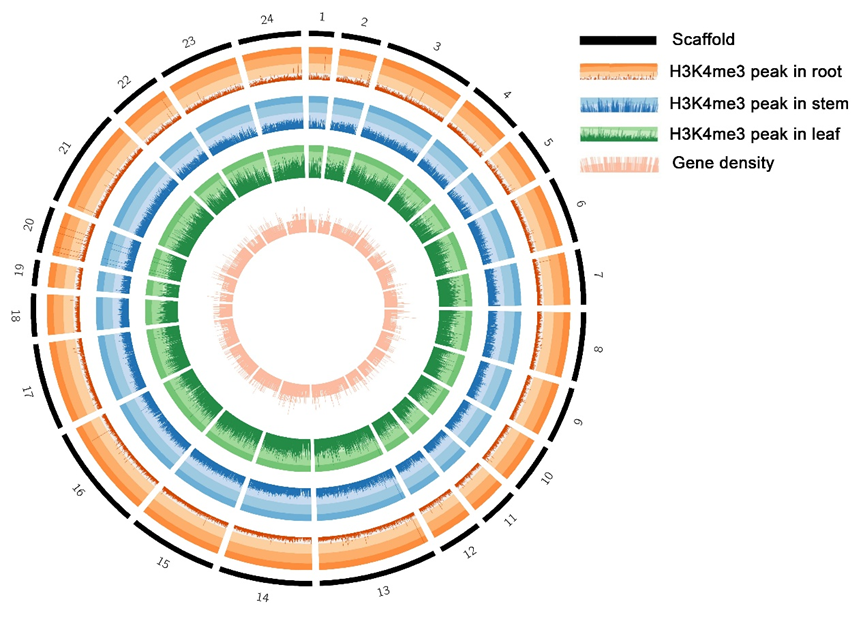


**Figure S1. Genome-wide distribution of H3K4me3 peaks and gene density.** The histone modification peaks of H3K4me3 in leaf (green), stem (blue), and root (orange) tissues, and gene density (pink) distribution along the whole bamboo genome version 2.0 including 24 scaffolds. In the calculation of gene density, 10,000 bp is used as a unit. The peak height indicates the enrichment score of H3K4me3.


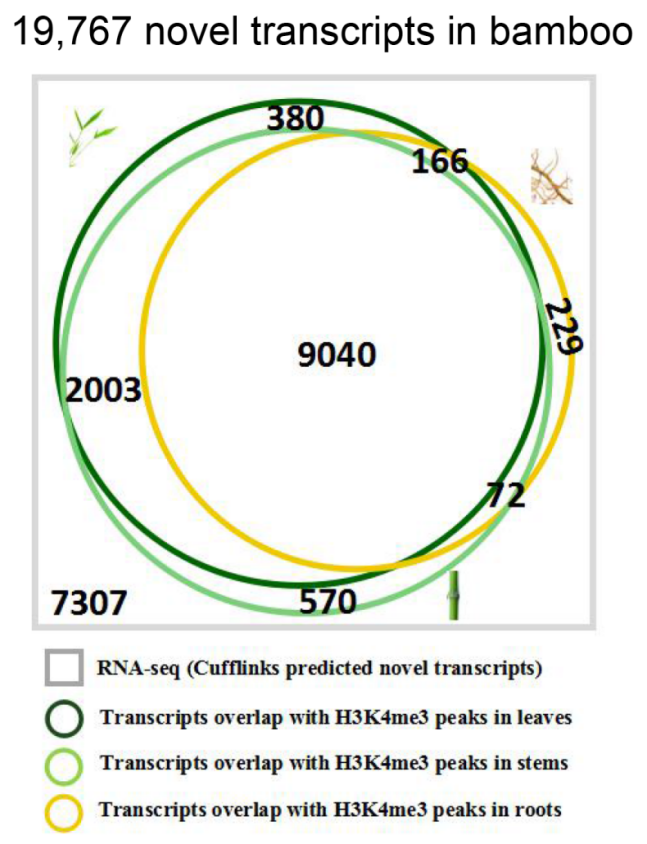


**Figure S2. The overlapped number of total novel transcripts in bamboo derived from RNA-seq datasets with H3K4me3 deposited peak regions in leaves, stems and roots.**


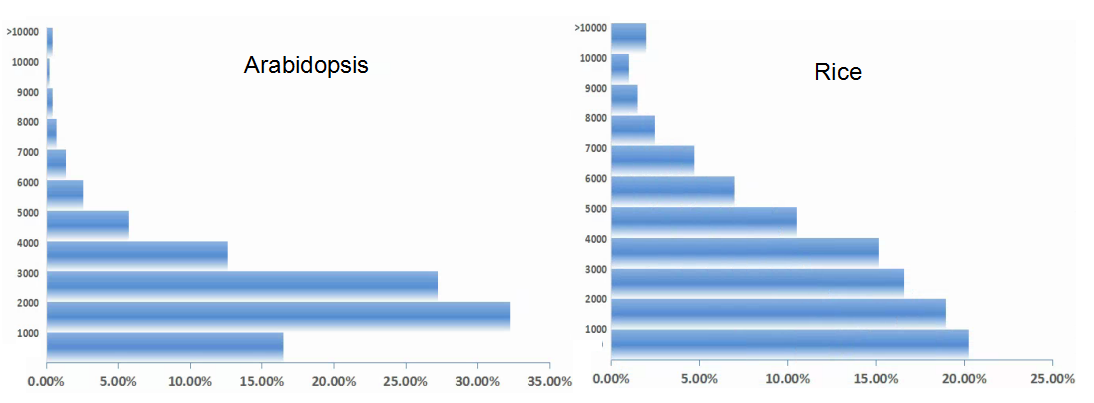


**Figure S3. The transcript length distribution of Arabidopsis (left) and rice (right).**


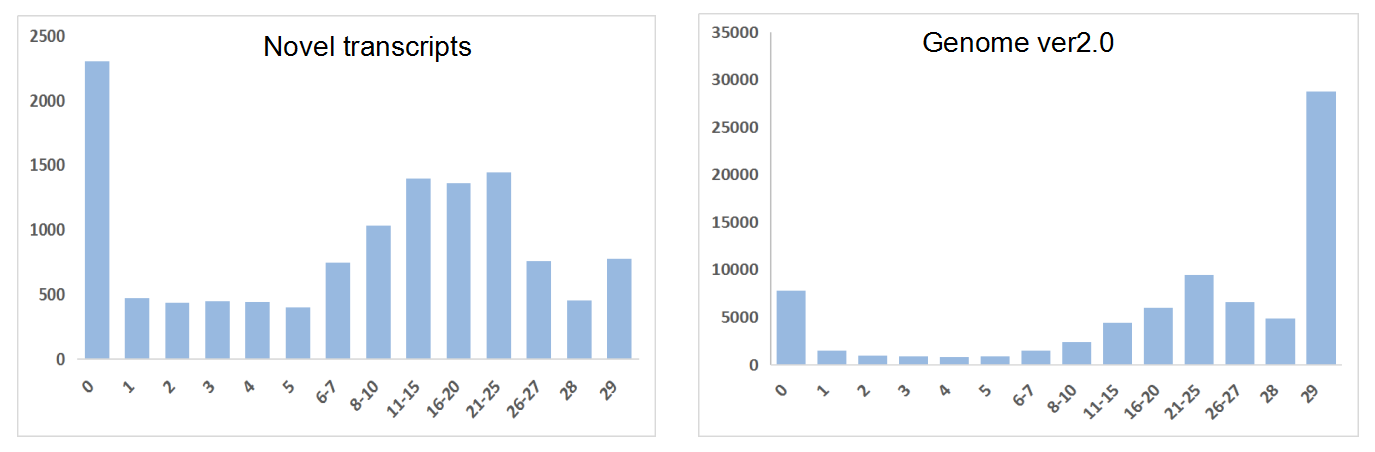


**Figure S4. The number of expressed transcripts in this prediction (left) and the latest annotated bamboo genome (right) among the number of the transcriptomic datasets.**


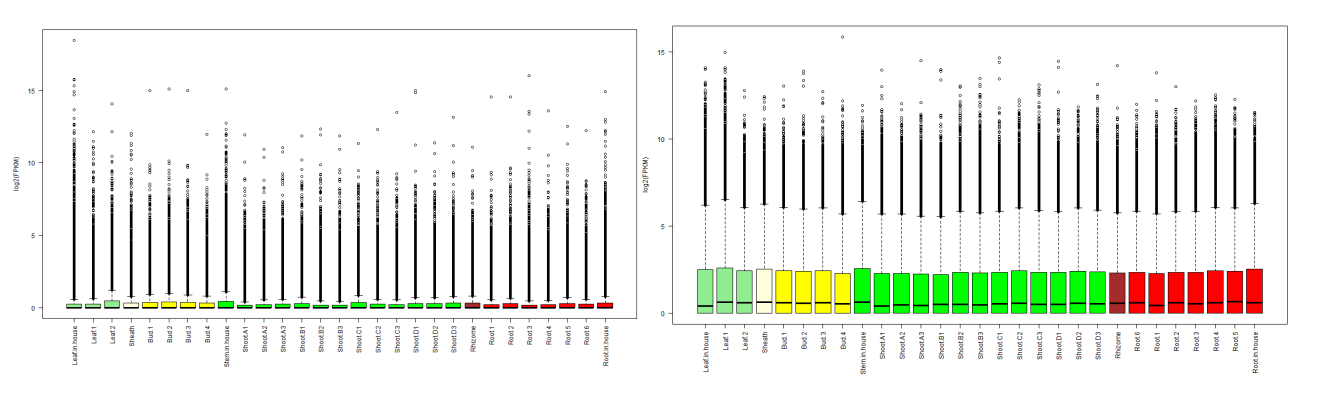


**Figure S5. The boxplot of expression value distribution in novel transcripts (left) and the latest annotated bamboo genome (right) in 29 transcriptomic datasets.**


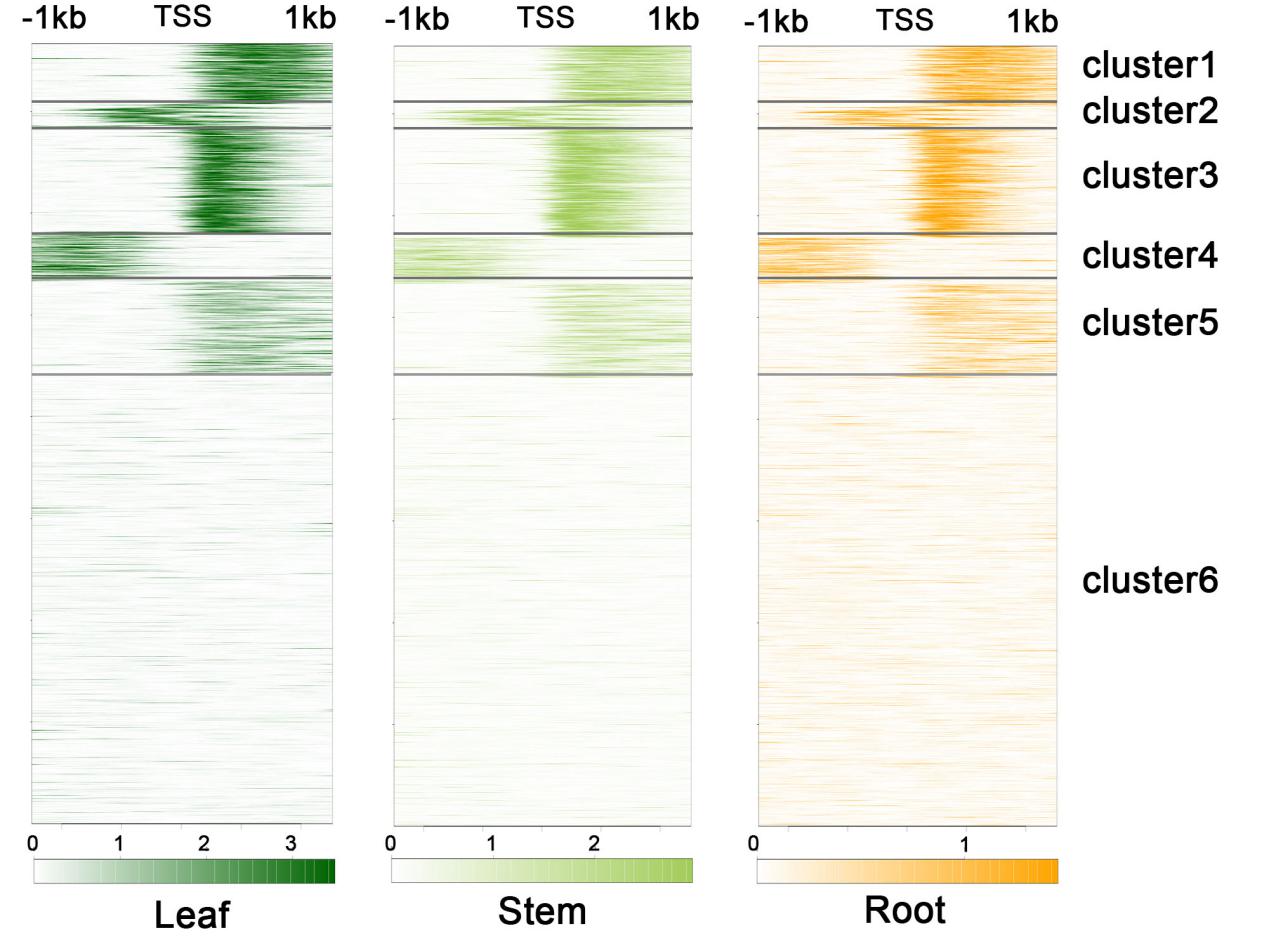


**Figure S6. Heat map of H3K4me3 around the TSSs of all of the bamboo transcripts and novel predicted transcripts in leaf, stem and root** (from 1 kb upstream to 1 kb downstream regions of the TSSs). All of these transcripts were clustered using the k-means method on the basis of their enrichment of histone mark H3K4me3 around the TSSs.


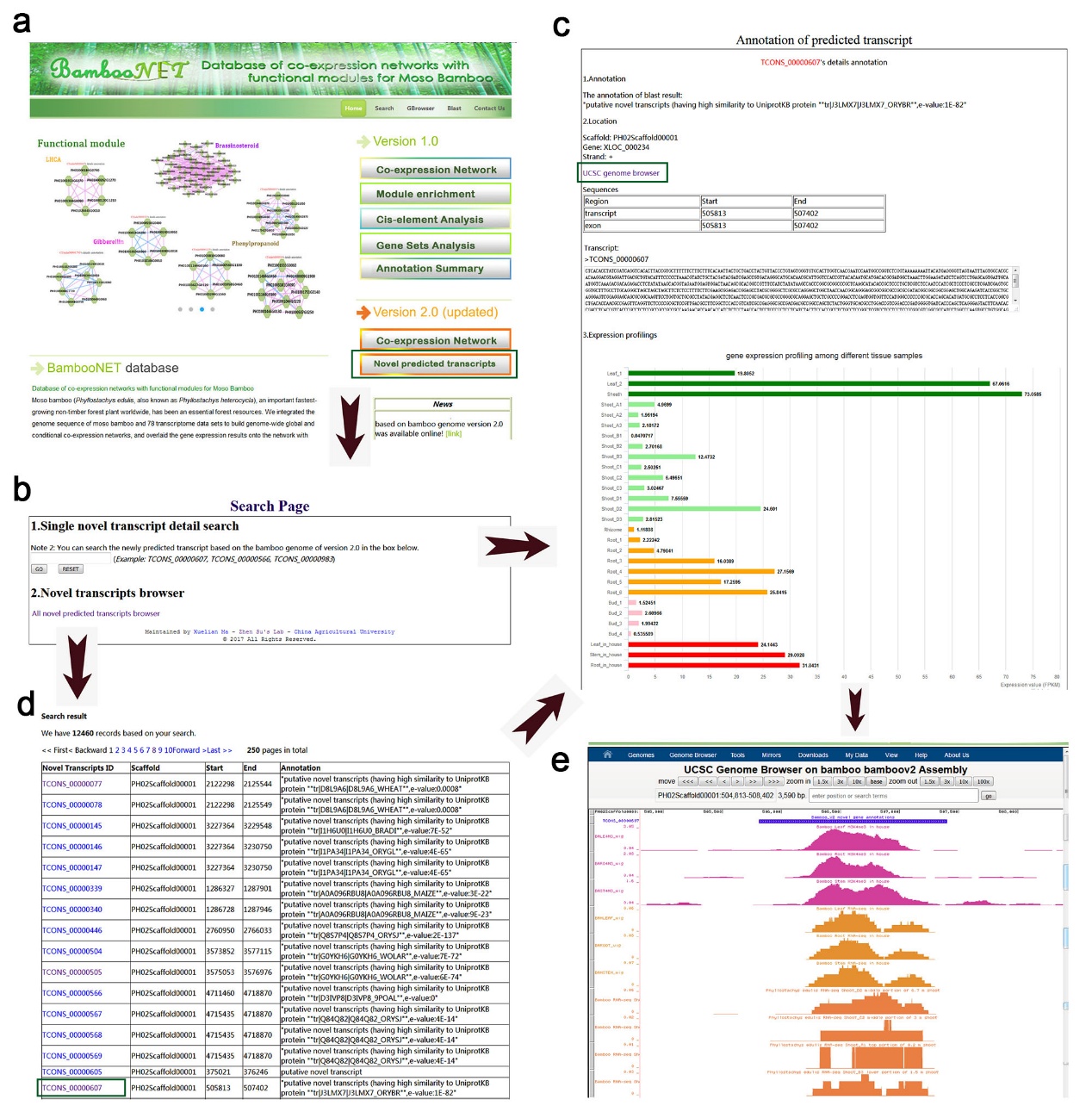


**Figure S7. General description of novel transcripts in the website.** (a) The option of “Novel predicted transcripts” shown in the homepage. (b) The search page of novel transcripts. (c) Basic information for novel transcript TCONS_00000607 obtained by using the search function, including annotation, location, and expression profiling in various tissues. (d) All novel predicted transcripts’ list by the link of the search page. (e) The novel transcript TCONS_00000607 shown in the UCSC Genome Browser.

**Supplemental Table 1. The mapping information of RNA-seq data of bamboo leaves, stems and roots tissues.**

| Sample | | | Bamboo_Leaf | Bamboo_Stem | Bamboo_Root |
| --- | --- | --- | --- | --- | --- |
| Name | | | BAMLEAF | BAMSTEM | BAMROOT |
| Reference genome | | | Bamboo ver2.0 | | |
| Left reads | Overall | Input | 23612119 | 23537521 | 23641319 |
|  |  | Mapped reads | 21030711 | 20935812 | 21086733 |
|  |  | Mapped rate  （of input） | 89.1% | 88.9% | 89.2% |
|  | Multiple | Mapped reads | 1230021 | 1099810 | 1108519 |
|  |  | Mapped rate | 5.8% | 5.3% | 5.3% |
| Right reads | Overall | Input | 23612119 | 23537521 | 23641319 |
|  |  | Mapped reads | 19852372 | 19731948 | 20033106 |
|  |  | Mapped rate  （of input） | 84.1% | 83.8% | 84.7% |
|  | Multiple | Mapped reads | 1145287 | 1026184 | 1044397 |
|  |  | Mapped rate | 5.8% | 5.2% | 5.2% |
| Overall read mapping rate | | | 86.6% | 86.4% | 87.0% |
| Aligned pairs | | | 18787432 | 18589402 | 18967103 |
| Concordant pair alignment rate | | | 78.6% | 77.9% | 79.5% |

**Supplemental Table 2. The mapping information of H3K4me3 ChIP-seq datasets of rice leaves, stems, and roots tissues.**

| Histone  modification | Species | Source | Tissue | | Total reads | Overall  alignment  rate | Aligned  exactly  1 time | Aligned >1  times | Peaks | Genes with peaks |
| --- | --- | --- | --- | --- | --- | --- | --- | --- | --- | --- |
| H3K4me3 | Rice | SRR6510882 | | Leaf | 13076082 | 97.41% | 76.08% | 16.20% | 26199 | 25334 |
|  |  | SRR6510894 | | Root | 31420450 | 91.17% | 65.03% | 15.24% | 32820 | 30873 |

**Supplemental Table 3. The summary of novel transcripts in annotated bamboo genome with transcriptomic datasets and epigenetic maps.**

| **Tissues** | **RNA-seq (Cufflinks predicted novel transcripts)** | **Transcripts overlap with H3K4me3 peaks in leaves** | **Transcripts overlap with H3K4me3 peaks in stems** | **Transcripts overlap with H3K4me3 peaks in roots** | **Transcripts overlap with H3K4me3 peaks in any tissue** |
| --- | --- | --- | --- | --- | --- |
| Rhizome | 8681 | 5763 | 5847 | 4899 | 6076 |
| Bud-4 (bud on rhizome) | 8878 | 5626 | 5714 | 4804 | 5954 |
| Root-6 (root on rhizome) | 7298 | 5174 | 5257 | 4496 | 5440 |
| Root-1 (0.1 cm root on shoot) | 7329 | 4769 | 4834 | 4144 | 5006 |
| Root-2 (0.5 cm root on shoot) | 7713 | 5502 | 5597 | 4766 | 5789 |
| Root-3 (2 cm root on shoot) | 6362 | 4720 | 4810 | 4127 | 4954 |
| Root-4 (10 cm root on shoot) | 6900 | 5020 | 5091 | 4357 | 5240 |
| Root-5 (new root with lateral roots) | 7559 | 5363 | 5436 | 4677 | 5633 |
| Shoot-A1 (top portion of 0.2 m shoot) | 7367 | 4590 | 4642 | 3952 | 4850 |
| Shoot-A2 (middle portion of 0.2 m shoot) | 7379 | 4830 | 4915 | 4181 | 5091 |
| Shoot-A3 (lower portion of 0.2 m shoot) | 7824 | 5223 | 5297 | 4490 | 5502 |
| Shoot-B1 (top portion of 1.5 m shoot) | 7257 | 5160 | 5276 | 4495 | 5432 |
| Shoot-B2 (middle portion of 1.5 m shoot) | 7015 | 4951 | 5058 | 4252 | 5218 |
| Shoot-B3 (lower portion of 1.5 m shoot) | 6257 | 4441 | 4544 | 3870 | 4689 |
| Shoot-C1 (top portion of 3 m shoot) | 6829 | 5079 | 5172 | 4402 | 5305 |
| Shoot-C2 (middle portion of 3 m shoot) | 7126 | 5142 | 5246 | 4439 | 5400 |
| Shoot-C3 (lower portion of 3 m shoot) | 7513 | 5235 | 5319 | 4527 | 5495 |
| Shoot-D1 (top portion of 6.7 m shoot) | 8287 | 5996 | 6125 | 5146 | 6304 |
| Shoot-D2 (middle portion of 6.7 m shoot) | 7084 | 5017 | 5114 | 4308 | 5272 |
| Shoot-D3 (lower portion of 6.7 m shoot) | 9214 | 5969 | 6061 | 5185 | 6316 |
| Leaf-1 (blade) | 6977 | 5099 | 5145 | 4297 | 5347 |
| Leaf-2 (leaf sheath) | 8462 | 5779 | 5877 | 4990 | 6120 |
| Sheath (Sheath sheet) | 9090 | 5857 | 5922 | 4891 | 6197 |
| Bud-1 (bud on top portion of 3 m shoot) | 9107 | 6316 | 6440 | 5369 | 6652 |
| Bud-2 (bud on middle portion of 3 m shoot) | 8355 | 6088 | 6244 | 5203 | 6422 |
| Bud-3 (bud on lower portion of 3 m shoot) | 8521 | 6065 | 6195 | 5178 | 6381 |
| Leaf (in house) | 5935 | 4443 | 4469 | 3826 | 4588 |
| Stem (in house) | 7434 | 5383 | 5472 | 4641 | 5611 |
| Root (in house) | 6751 | 4906 | 4951 | 4302 | 5095 |
| total | 19767 | 11589 | 11685 | 9507 | **12460** |

**Supplemental Table 4. A list of primers for semiquantitative RT-PCR and qRT-PCR used in this study**

| Type | | Target | Forward | Reverse |
| --- | --- | --- | --- | --- |
| PCR | TCONS_00131779 | | CGGTATCTGATCGTCTTCG | TTTGTTTGATGGTACGTGCTA |
|  | TCONS_00025830 | | TACTGTTGTGCGCGTGTGGCT | TGCCGAGTATAAGCACGGGAT |
|  | TCONS_00043481 | | GATGAGGCAGAGATCTAAGT | TTGTCTAGAGTGCAAAACTCT |
|  | TCONS_00079011 | | GAGAAGGGGAGCGCTTGTATC | GCATACATAATTATCATTAATTATTAG |
|  | TCONS_00030666 | | CCCATCCCCTCGCCCCGC | ATGTGCGATTAACTCATGTTCAA |
|  |  | |  |  |
| qRT-PCR | TCONS_00131779 | | ACCTACTTGACCCTTCTG | CTTTGAGCACTCTAATTTCTTC |
|  | TCONS_00025830 | | GTCGCCTTCGGGATCTAC | ACCTAGTGGTGGTGGTCG |
|  | TCONS_00043481 | | TATTGCTACCCATGTCTT | ATAAGCCACTCAACAGAT |
|  | TCONS_00079011 | | CTAAAGACTGAGGAGGAT | GGACTGTATGACATAAGAC |
|  | TCONS_00030666 | | CTAAAGACTGAGGAGGAT | GGACTGTATGACATAAGAC |
|  | 18SrRNA | | CGGCTACCACATCCAAGGAA | TGTCACTACCTCCCCGTGTCA |

**Supplemental Table 5. The distribution of Arabidopsis genes with exon numbers**

| **Exon number/gene** | **Percentage** |
| --- | --- |
| 1 | 26.36% |
| 2 | 12.91% |
| 3 | 9.96% |
| 4 | 8.21% |
| 5 | 7.32% |
| 6-7 | 11.01% |
| 8-10 | 10.86% |
| 11-15 | 8.72% |
| >16 | 4.66% |

**Supplemental Table 6. The distribution of rice genes with exon numbers**

| **Exon number/gene** | **Percentage** |
| --- | --- |
| 1 | 1.94% |
| 2 | 5.29% |
| 3 | 7.90% |
| 4 | 8.08% |
| 5 | 8.14% |
| 6 | 7.60% |
| 7 | 7.38% |
| 8 | 6.80% |
| 9 | 6.11% |
| 10 | 5.56% |
| 11 | 5.04% |
| 12 | 4.74% |
| 13 | 3.95% |
| 14 | 3.38% |
| 15-16 | 5.24% |
| 17-19 | 5.42% |
| 20-25 | 5.14% |
| >25 | 2.30% |
